# Polymicrobial infections with specific *Actinomyces* and related organisms, using the current taxonomy

**DOI:** 10.1080/20002297.2024.2354148

**Published:** 2024-05-16

**Authors:** Eija Könönen

**Affiliations:** Institute of Dentistry, University of Turku, Turku, Finland

**Keywords:** *Actinomyces*, *Gleimia*, *Schaalia*, *Winkia*, taxonomy, commensal, pathogen, polymicrobial infection, clinical relevance

## Abstract

*Actinomyces* organisms reside on mucosal surfaces of the oropharynx and the genitourinary tract. Polymicrobial infections with *Actinomyces* organisms are increasingly being reported in the literature. Since these infections differ from classical actinomycosis, lacking of specific clinical and imaging findings, slow-growing *Actinomyces* organisms can be regarded as contaminants or insignificant findings. In addition, only limited knowledge is available about novel *Actinomyces* species and their clinical relevance. The recent reclassifications have resulted in the transfer of several *Actinomyces* species to novel genera *Bowdeniella*, *Gleimia*, *Pauljensenia*, *Schaalia*, or *Winkia*. The spectrum of diseases associated with specific members of *Actinomyces* and these related genera varies. In human infections, the most common species are Actinomyces *israelii*, *Schaalia meyeri*, and *Schaalia odontolytica*, which are typical inhabitants of the mouth, and *Gleimia europaea*, *Schaalia turicensis*, and *Winkia neuii*. In this narrative review, the purpose was to gather information on the emerging role of specific organisms within the *Actinomyces* and related genera in polymicrobial infections. These include *Actinomyces graevenitzii* in pulmonary infections, *S. meyeri* in brain abscesses and infections in the lower respiratory tract, *S. turicensis* in skin-related infections, *G. europaea* in necrotizing fasciitis and skin abscesses, and *W. neuii* in infected tissues around prostheses and devices. Increased understanding of the role of *Actinomyces* and related species in polymicrobial infections could provide improved outcomes for patient care.

Key messages

- Due to the reclassification of the genus, many former *Actinomyces* species belong to novel genera *Bowdeniella*, *Gleimia*, *Pauljensenia*, *Schaalia*, or *Winkia*.
- Some of the species play emerging roles in specific infection types in humans.
- Increasing awareness of their clinical relevance as an established or a putative pathogen in polymicrobial infections brings about improved outcomes for patient care.

## Introduction

Many *Actinomyces* taxa are considered human pathogens despite of being part of the commensal microbiome at various sites of the human body. The causative role of *Actinomyces israelii* (*Streptothrix israeli*) in classical actinomycosis is known since the late nineteenth century [1]. In this endogenous infection, the etiological gram-positive organism gains access to deeper tissues via trauma, surgical procedures, or foreign bodies, thus disrupting the mucosal barrier, and when inside the tissue, typical branching, filamentous *Actinomyces* cells form bacterial aggregates [2,3]. The presence of hard ‘sulfur granules (grains)’ are considered confirmatory characteristics for actinomycotic lesions. Actinomycosis is a chronic disease, proceeding slowly and forming local abscesses with sinus tracts and pus secretion. A recent PubMed search with the key word ’actinomycosis’ revealed over 8.300 articles, but around half of them are case reports where, notably, diagnosis is often based only on clinical findings with rather unspecific general symptoms like cough and fever and on histopathological samples (but where hard ‘sulfur granules’ can be absent). It is noteworthy that when microbiological diagnostics is missing, the causative agent remains unknown.

Only a minority of infections caused by *Actinomyces* organisms in humans seems to possess classical characteristics of actinomycosis with specific clinical, imaging, and histological findings. It is likely that considerable part of infectious cases where *Actinomyces* organisms are involved lack typical actinomycotic lesions, and instead, are other types of infections [2]. When the indicated species is present as a member of polymicrobial infectious consortia, like in abscesses at various locations, its clinical relevance may be underestimated due to challenges in recognizing its involvement in polymicrobial infections. Since advanced methods are now available for bacterial detection and identification in clinical microbiology laboratories, this improves the species-level information and gradually accumulates knowledge about their clinical significance. In a comprehensive review on *Actinomyces* and related organisms [2], the gathered literature indicated that there are ‘preferred’ body sites and infection types where individual *Actinomyces* species seem to play an important role.

Recently, Bartlett et al. [4] published an extensive search for human bacterial pathogens described before 2021. A bacterial species was dedicated as pathogenic, if isolated either at the site of symptomatic infection or when with a toxin-mediated illness acting at another site. Pathogens were defined either as ‘established’ (at least three references relating to three or more infected individuals) or ‘putative’ (less than three known cases). In other words, the species in the latter category need for further reports until the confirmation of their established pathogenicity. Notably, among the top-10 bacterial genera housing most pathogen species was *Actinomyces*, with 16 established and 10 putative pathogen species [4].

The aims of the present narrative review are to give an overview on *Actinomyces* organisms in polymicrobial infections, considering their changing taxonomy, and to focus on a few species playing emerging roles in specific infection types in humans.

## Current taxonomy and challenges in detecting clinical relevance of the taxa

In the twenty-first century, the *Actinomyces* genus expanded considerably, doubling the number of validly published *Actinomyces* species colonizing humans [2]. A recent genome-scale taxonomic analysis performed by Nouioui et al. [5] determined the phylogenetic position of the phylum Actinobacteria (currently *Actinomycetota* [6]), and this resulted in five novel genera, *Bowdeniella, Gleimia*, *Pauljensenia*, *Schaalia*, and *Winkia*, hosting part of former *Actinomyces* species isolated from human clinical specimens. Table 1 presents the validly published species with changes in their taxonomy; the human species within the five novel genera currently include *Bowdeniella nasicola, Gleimia europaea* and *G. hominis*, *Pauljensenia hongkongensis*, *Schaalia cardiffensis*, *S. funkei*, *S. georgiae*, *S. meyeri*, *S. odontolytica*, *S. radingae*, and *S. turicensis*, and *Winkia neuii* with its two subspecies *anitrata* and *neuii* [5,7]. In addition to this expansion and reclassifications, a plethora of species-level taxa is waiting to be characterized and/or approved (https://lpsn.dsmz.de/search?word=actinomyces).

**Table 1.** Updated taxonomy of validly published *Actinomyces* species in humans [5,7].

Former *Actinomyces* sp.	Current taxonomy
*A. nasicola*	*Bowdeniella nasicola*
*A. europaeus*	*Gleimia europaea*
*A. hominis*	*Gleimia hominis*
*A. hongkongensis*	*Pauljensenia hongkongensis*
*A. cardiffensis*	*Schaalia cardiffensis*
*A. funkei*	*Schaalia funkei*
*A. georgiae*	*Schaalia georgiae*
*A. meyeri*	*Schaalia meyeri*
*A. odontolyticus*	*Schaalia odontolytica*
*A. radingae*	*Schaalia radingae*
*A. turicensis*	*Schaalia turicensis*
*A. neuii*	*Winkia neuii*
subsp. *neuii*	subsp. *neuii*
subsp. *anitratus*	subsp. *anitrata*

It is worth mentioning that most of the *Actinomyces* species described in the twenty-first century are based on a single strain or a few strains from clinical specimens. Therefore, it is unclear what is their habitat/source, and since some species grow slowly, forming tiny, undistinguished colonies, they may easily remain unnoticed in clinical samples. Furthermore, many species within *Actinomyces* and related genera differ from a typical branching-rod cell morphology of *A. israelii*; for example, *A. radicidentis* has coccoid cells [8], while some species like *G. europaea* and *S. meyeri* are short rods [9,10], and *W. neuii* with its two subspecies appear as diphteroidal, non-branching rods [11]. These atypical morphologies may result in failure to identify them as being *Actinomyces* organisms in direct Gram stain of the specimen, leading to a misinterpretation as ‘normal flora contaminants’. Thus, their potential involvement in different pathologies may be unrecognized and, in such way, the data in the literature accumulate slowly.

## Detection and impact of commensals as infecting agents

Members of *Actinomyces* and related genera are considered commensals at different body sites, their major habitats being the oral cavity, pharynx, distal esophagus, and the genitourinary tract [2]. However, they are also capable of acting as indigenous causative agents in various types of human infections more or less from head to toe.

Species within the genera *Actinomyces* and *Schaalia*, in particular, are among the common colonizers of the human mouth where *A. naeslundii* and *A. oris* are known as basic components of dental biofilms [12]. In our previous studies on the development of oral and nasopharyngeal anaerobic microbiotas in young children, a strict longitudinal follow-up was designed for collecting saliva and nasopharyngeal samples from 50 infants aged 2 months at baseline, and again in scheduled visits four times continuing up to 2 years of age [13,14]. For *Actinomyces*, comprehensive culture techniques were used and an advanced identification flowchart, including a variety of phenotypic, enzymatic, and fermentation reactions, was developed [15]. We found *S. odontolytica* in saliva of 30% of the predentate infants at baseline. Unlike *S. odontolytica*, most *Actinomyces* species did not appear until tooth eruption; however, a classical pathogen *A. israelii* was mainly absent during the follow-up. We made a special effort to detect the newly described *Actinomyces* species, but it seems that many of them are rare colonizers in the oral cavity or establish later in life, if ever. Interestingly, this study was the first to demonstrate *A. graevenitzii* as an oral species [13]. Nasopharyngeal swab samples were collected from the same infant cohort during scheduled visits, and nasopharyngeal aspirate samples, whenever acute otitis occurred; *Actinomyces* was a rare finding during health, but frequently isolated from aspirate samples collected during otitis episodes [14]. Unfortunately, in that study *Actinomyces* recoveries from the nasopharynx were not identified to the species level.

*A. gerencseriae*, *A. graevenitzii*, *A. israelii*, *A. naeslundii*, *A. oris*, and *S. georgiae*, *S. meyeri*, and *S. odontolytica* are common oral residents within the genera *Actinomyces* and *Schaalia*, respectively, and also *A. massiliensis* and *A. timonensis* from the oral cavity and *A. massiliensis*, *A. radicidentis*, and *S. cardiffensis* from the pharynx have been reported [2,16,17]. In the distal esophagus, the microbiota has shown to be similar to that of the oral cavity, among the species recovered are *S. odontolytica*, *S. meyeri*, and *A. graevenitzii* [18]. It was speculated whether oral bacteria selectively passage from the oropharynx or whether this is due to a selective retention of particular species by the esophagus. *Actinomyces* and *Schaalia* are abundant in the oral cavity/oropharynx, and are a source not only for oral but also for non-oral infections when translocating via tissue invasion or via bloodstream to other body sites.

The female genitourinary tract is another common location for the colonization of *Actinomyces* organisms, *A. urogenitalis*, *S. meyeri*, *S. radingae*, *S. turicensis*, and *W. neuii* being present without connection to infectious processes [2]. However, *S. turicensis* and *W. neuii*, in particular, are increasingly detected in polymicrobial infections at lower body sites.

Indeed, commensals are often isolated from clinical specimens together with other bacteria [2]. Utilizing laboratory-based criteria and clinical parameters, Leal et al. [19] analyzed retrospectively data on coryneform gram-positive bacilli in various types of specimens collected at an academic medical center in North Carolina from years 2012 to 2015, for evaluating whether the findings represented true infection or contamination. Among the 18% findings deemed to be clinically significant, several *Actinomyces* taxa were found and came typically from polymicrobial abscesses, cysts, or seromas [19]. The authors underlined the potential impact of commensal bacteria in specific types of infections and recommended a species-level identification of *Actinomyces* isolates. In a study from London, UK, clinical significance of *Actinomyce*s found in blood specimens was evaluated retrospectively from NHS Trust records between October 2009 and December 2014 [20]. Most blood isolates from 60 patients were *S. odontolytica*. Ten of the patients who had received treatment (prolonged antibiotic therapy and/or surgery) due to pulmonary, abdominal, dental, or disseminated actinomycosis, soft tissue disease, or disease not categorized were compared to 50 patients positive for *Actinomyces* in blood culture but without need for treatment. No apparent negative impact on clinical outcomes was observed between the treated and untreated groups. The authors speculated whether *Actinomyces* could be blood culture contaminants or represent transient bacteremia by commensals translocated from their habitats to blood [20]. Moreover, Lynch et al. [21] examined 115 invasive infections with involvement of *Actinomyces*, diagnosed in a Canadian health care region between 2011 and 2014. In a variety of severe infections, such as pulmonary, bone, central nervous system, and bloodstream infections, and skin and soft tissue abscesses, *Actinomyces* was considered the principal pathogen. Among the abundant isolates, 16S rRNA sequencing revealed many still un-named oral taxons available in public databases [21]. The recognition of commensal bacteria as being clinically relevant findings, instead of interpreting them as contaminants, can be challenging without specific efforts by clinical microbiology laboratories.

Advanced methods like matrix-assisted laser desorption ionization – time of flight mass spectrometry (MALDI-TOF MS) and/or sequencing of the 16S rRNA gene have widely replaced laborious culture techniques and often complex biochemical methods [15] for identifying and separating different *Actinomyces* organisms. In testing of MALDI-TOF MS for identifying of oral *Actinomyces* species, Stingu et al. [22] underlined the importance of including a sufficient number of strains in the reference database when assigning less common *Actinomyces* species (here: *A. graevenitzii*, *A. radicidentis*, *A. urogenitalis*, *G. europaea*, *S. georgiae*, and *W. neuii*) and recommended to confirm their identification by 16S rRNA sequencing. Although these advanced methods are now routinely utilized in well-equipped clinical microbiology laboratories for recognizing the potential pathogen(s), a direct Gram stain should be examined at the time of plate reading when an infectious role of commensals is suspected (e.g. predominant growth from wound samples), since polymicrobial infections complicate the interpretation of the results [19]. As shown by Leal et al. [19], a considerable proportion of diphteroid-like isolates proves to be clinically significant; thus, they suggested all diphteroid-like isolates, including *Actinomyces*, to be identified to the species level, or in the case of *W. neuii*, even to the subspecies level via MALDI-TOF MS or 16S rRNA sequencing. The species-level identification could support clinicians’ decision-making, for example, in the estimation whether the finding represents true infection or contamination and whether its antimicrobial susceptibility pattern needs attention. A study by Fong et al. [23] evaluated the performance of MALDI-TOF MS for the identification of *Actinomyces*, using 16S rRNA gene sequencing as the reference method. Moreover, they examined the impact of MALDI-TOF MS in clinical microbiology laboratories on elucidating the diversity of *Actinomyces* species in infections at different body sites. Compared to pre-MALDI-TOF era, the method increased the detection rates of *Actinomyces* organisms, identifying nearly 60% of the 77 isolates to the species level and nearly 90% to the genus level. The top-three most important species in clinical samples were, in descending order, *S. turicensis, S. odontolytica*, and *W. neuii*, followed by *A. oris*, *A. naeslundii*, and *G. europaea* [23]. On the other hand, in a Canadian laboratory, where 115 *Actinomyces* isolates from invasive infectious cases were identified using partial 16S rRNA sequencing and MALDI-TOF MS, the ability of the latter method to identify the diverse array of *Actinomyces* species was poor, as only less than half (41%) of the isolates was correctly identified [21]. There seem to be differences in the performance of commercial MALDI-TOF systems to identify *Actinomyces* and related genera to the species but also to the genus level [24]. To expand the spectral databases to be complete enough is warranted to guarantee a reliable detection of less known *Actinomyces* taxa [21,24].

## Typical infectious pattern for specific *Actinomyces* and related taxa

During the 1990s, many novel *Actinomyces* species of clinical interest were described, gradually changing the role of these species from contaminants to infectious agents. Some dedicated microbiology researchers became interested in elucidating the association of individual *Actinomyces* species with disease at specific sites [25–27]. Since only a limited number of strains had been examined, there was an obvious lack of knowledge about their habitats and pathogenic potential as well as connection to specific infections. Moreover, routine diagnostic methods used in clinical microbiology laboratories at that time were not sufficient to reach an accurate identification and species separation within the *Actinomyces* genus. Due to the availability of molecular diagnostic methods in pioneer microbiology laboratories, the understanding of the pathogenic role of *Actinomyces* started to increase.

Among a large collection of clinical *Actinomyces*-like isolates from Belgium, Sabbe et al. [25] identified a vast majority of them as *S. turicensis*, and some isolates were identified as *S. radingae* and *G. europaea*, which all represented newly described *Actinomyces* species at that time [9,11,28]. The genitourinary tract was the major site infected with *S. turicensis*, while another typical location for infections was connected to the skin and soft tissues, mostly below the waistline, where these three species were clinically relevant findings [25]. Two years later, Hall et al. [26] reported the results of their analyses of the incidence and clinical associations of over 400 *Actinomyces* isolates collected in English and Welsh hospital laboratories from 1983 to 1999. *S. turicensis* and *A. israelii* proved to be the most common species in clinical samples, followed by *A. naeslundii*, *S. odontolytica*, and *A. gerencseriae*. The majority of *A. israelii* as well as *A. gerencseriae, A. naeslundii*, and *S. odontolytica* originated from intrauterine device and neck-face infections, while the origin of *S. turicensis* was the urogenital tract, and it was detected in soft tissue lesions, mainly at the lower part of the body [26], confirming similar findings on *S. turicensis* from Belgium [25]. Microbiological data from Texas [27] included 100 putative *Actinomyces* isolates from human infections to be sequenced, and again, *S. turicensis* was the most frequent species with a similar disease pattern observed in the two previous studies [25,26]. In some cases, *S. turicencis* grew in pure culture, indicating its pathogenicity [25,27].

Increasing data on members of *Actinomyces* and related genera from clinical microbiology laboratories reported in the literature strengthens the concept of preferred infectious sites for different species [2]. A similar observation was made by Lynch et al. [21] confirming that some *Actinomyces* organisms have a tendency to associate with specific types of infection; for instance, *S. radingae* and *W. neuii* occurred in skin and soft tissue abscesses, and *S. odontolytica* in bloodstream infections, while *A. graevenitzii* was connected to pulmonary infections only. In addition, among the sequencing findings were several un-named oral taxa in pulmonary fluids, infectious bone specimens, and blood [21].

### Actinomyces graevenitzii

Indeed, *A. graevenitzii* is an oral species and its infectious recoveries come almost exclusively from respiratory sites [29]. In this context, a proper protection of bronchial specimens from contamination by commensals is important to avoid distorted interpretations, as was shown for *A. graevenitzii* in an unusual pseudo-outbreak at a university-affiliated teaching hospital [30]. An interesting difference was noticed in a metagenomic analysis of the tongue microbiome where *A. graevenitzii* was one of the five species having distinct single-nucleotide variant profiles between current and never smokers [31]. In the lung microbiome of lung cancer patients, who are often smoking males, *A. graevenitzii* was reported to relate to squamous cell carcinoma but not to adenocarcinoma [32]. Furthermore, in stroke-associated pneumonia, certain oral members of the phylum *Actinomycetota* may have a significant influence on the outcome [33]; their enrichment was shown as an independent risk factor, being related to functional poor outcomes within 30 days, while *Streptococcus* proved to have a protective effect. A meta-analysis including five studies compared the composition of the lower respiratory microbiota between tuberculosis patients and their healthy controls; a distinct abundance signature co-occurring with *Mycobacterium tuberculosis*, among those *A. graevenitzii* and *Rothia mucilaginosa* as driving forces towards tuberculosis was identified [34].

### Schaalia meyeri

The disease pattern of *S. meyeri* has drawn increasing attention as being connected to infectious processes at intracranial and pulmonary sites. Already in the original description of *S. meyeri* including 16 strains [10], four of the strains originated from intracranial abscesses and three strains from lung specimens. Among polymicrobial consortia of brain abscesses, *S. meyeri* is the most frequently reported *Actinomyces* organism [21,35–38], in many cases with *Aggregatibacter aphrophilus*, *Fusobacterium nucleatum*, *Parvimonas micra*, and/or anginosus group streptococci [35,36,38–41]. Since they all are common oral findings, it is not surprising that a dental background is often highlighted. Recently, the nationwide Danish Study Group of Infections of the Brain (DASGIB) published a population-based study, covering all adult-aged patients with brain abscess positive for typical oral bacteria from 2007 to 2020, aiming to the incidence, clinical presentation, and prognostic factors [42]. Of the 287 cases identified, 41% were polymicrobial, with similar bacterial findings previously reported, though presented only at the genus level. One-third had immunocompromised status, while one-fourth had dental infection and one-tenth upper respiratory infection, which were seen as risk factors for brain abscess. Of those, dental infection was associated with a decreased risk of unfavorable outcome [42]. After the observation of microbiological similarities between brain abscesses and pleural empyemas, Dyrhovden et al. [37] analyzed bacterial findings in 27 empyemas of poorly described etiology and compared them to those in 25 brain abscesses of assumed oral/sinus origin. According to their hypothesis, certain oral bacteria could expose to purulent infections elsewhere in highly oxygenated organs, including the brain and lung. Among the most common infectious findings in these organs were *F. nucleatum*, *P. micra*, anginosus group streptococci, but also *S. meyeri* [37]. In addition, a few case reports exist on *S. meyeri* recoveries from pleural infections [43,44]. To be infected with *S. meyeri*, various dental procedures, tooth extractions, and poor oral hygiene are seen as predisposing factors [35,39,43–47]. Knowledge of virulence factors of *Actinomyces* and related taxa is scarce, and why *S. meyeri* has a tendency for being involved in severe conditions at these distant sites is not clear so far.

### Schaalia turicensis

Evidence on *S. turicensis* as an important human pathogen in infectious processes present in soft tissues at lower body sites is consistently emerging [19,23]. Further support for its pathogenic role comes from a recent case series of 15 pilonidal and perianal infections [48]. A devastating infectious process from where *S. turicensis* has been recovered as one of the etiological organisms is Fournier’s gangrene, a destructive necrotizing fasciitis, in the perineal and genital regions [49,50]. In addition, *S. turicensis*, together with *Bacteroides thetaiotaomicron* and *Staphylococcus epidermidis*, was found in necrotizing fasciitis at cervicofacial sites with an odontogenic focus [51]. These reports indicate that atypical microorganisms like *S. turicensis* deserve to be taken into account as a potential player in the pathogenesis of Fournier’s gangrene. It seems that *S. turicensis* is increasingly recognized at sites located at the upper part of the body, such as brain abscess [52], breast abscesses [53,54], supraglottitis with deep neck space abscesses [55], pleural empyema fluid [56], and a deep shoulder infection after surgery where *S. turicensis* was the only microbial finding [57]. Also, a fatal case of meningitis has been reported as a complication of purulent mastoiditis, where *S. turicensis* was identified in both pus and cerebral fluid specimens [58]. In all these cases, *S. turicensis* was identified using MALDI-TOF MS or sequencing methods.

### Gleimia europaea

*G. europaea* is known to be involved in superficial soft tissue infections at both upper and lower body sites, and infections at the urogenital area [2,9,53]. As an example is a recent case on subcutaneous abscess at chest with a mixed infection of *G. europaea* with a gram-positive anaerobic coccus, *Peptoniphilus olsenii*, in a male patient suffering from COVID-19 and receiving immunosuppressant therapy [59]. Moreover, suppuration from infected keloid scars contains a variety of bacteria, including *G. europaea* [60]. According to recent case reports, *G. europaea* has now appeared as a potentially significant pathogen in necrotizing fasciitis, a severely progressive infection, destructing skin, subcutaneous tissue, muscle, and fascia. Its recognition might be due to an increased awareness of the role of previously unrecognized, atypical organisms. In the first report, linking this organism to necrotizing fasciitis, *G. europaea* was together with *Actinotignum schaalii* in an elderly diabetic patient with a history of urinary tract infections [61]. The second patient case found *G. europaea* as the primary causative agent of necrotizing fasciitis [62]. The authors of the third case described *G. europaea* as an emerging causative agent of this devastating infection [63]. Notably, the reduced antimicrobial susceptibility pattern of *G. europaea* needs attention. Further, a fulminant case diagnosed as Fournier’s gangrene was connected to *G. europaea* as the only causative agent [64]. An interesting observation is that, in all the four cases, the patients were diabetics with other comorbidities. As underlined in these reports, early detection of the causative agent(s) is of utmost importance for a proper treatment to reduce mortality.

### Winkia neuii

Similar to *S. meyeri*, *S. turicensis*, and *G. europaea*, the detection of *W. neuii* in clinical samples has become more frequent due to improved detection methods. In particular, its role in abscesses of the skin and soft tissues has been observed in several studies [19,21,53,65]. Among nearly 400 clinical *Actinomyces* isolates collected from deep abscesses in an Austrian tertiary care center within a seven-year period, *S. meyeri* and *S. turicensis* were predominant, accounting for 34% and 23% of the abscess recoveries, respectively [66]. Although *W. neuii* was less prevalent, it accounted for 8% of the isolates. In a study focusing on the role of diphteroids in clinical infections [19], *W. neuii*, in particular, and *S. turicensis* were reported as the most important *Actinomyces* organisms in wounds. *W. neuii* has been among infectious findings associated with breast implant, shoulder implant, or a peritoneal dialysis catheter [67–69]. In addition, *W. neuii* has been isolated from clinical specimens of the genitourinary tract [19,23,53,70].

## Remarks on pathogen status of *Actinomyces* and related taxa

To date, 16 species within the genera *Actinomyces*, *Gleimia*, *Schaalia*, or *Winkia* are characterized as established pathogens in humans [4]. While all human *Actinomyces* species described before 2000 are recognized as established pathogens, only four species described thereafter, including *A. oris*, *A. urogenitalis*, *S. funkei*, and *S. cardiffensis*, belong to the category of established pathogens. However, data on the clinical relevance of *A. urogenitalis*, *S. cardiffensis*, and *S. funkei* are still relatively scarce. *A. urogenitalis* is seen as an uropathogen [71], but only a few reports are available. Two bacteremic episodes caused by *A. urogenitalis* have been reported [72,73]. In addition to bacteremia, 11 *A. urogenitalis* strains isolated from clinically relevant samples in a university hospital came from bone/soft tissues, genital abscesses, or urine [53]. Eight strains of *S. cardiffensis* from a variety of sources (pus from ear, sinus washout, pus from jaw abscess, pleural fluid, intrauterine devices, and pericolic abscess) were available for its description by Hall et al. [74]. Since then, cases of bacteremia with liver and lung abscesses [75], severe organizing pneumonia with lung abscess [76], and brain abscesses [38,77] positive for *S. cardiffensis* have been reported. In addition to three *S. funkei* strains in the description paper [78], there are only some occasional findings in the literature; among those are an isolate from intraabdominal pelvic abscess [21], six isolates from abscesses, biopsy, or superficial wounds at lower body sites [79], and isolate from Fournier’s gangrene [80], found as part of polymicrobial infections.

As presented in Table 2, six oropharyngeal *Actinomyces* species, namely *A. dentalis* [84], *A. johnsonii* [83], *A. oricola* [85], and *A. radicidentis* [8] are considered putative pathogens in the bacterial pathogen list of Bartlett et al. [4], as do *A. massiliensis* [86], originally isolated from human blood, and *A. timonensis* [87], originally isolated from sacroiliitis specimen. In addition, two former *Actinomyces* species within the genera *Gleimia* and *Pauljensenia*, *G. hominis* [81] and *P. hongkongensis* [82], respectively, are putative pathogens. Instead, the former *A. nasicola* [88], which currently belongs as the only species to the novel genus *Bowdeniella* as *B. nasicola*, was characterized based on a single isolate from nasal antrum aspirate without a known habitat and is not listed as being a pathogen so far. It is obvious that the species in the category of putative pathogens need for further reports until the confirmation of their established pathogenicity.

**Table 2.** *Actinomyces* and related taxa not-yet characterized as established pathogens in humans according to Bartlett et al. [4].

Species	Year of description [reference]	Isolation site(s) (no. of strains^a^)	Pathogen status
*A. radicidentis*	2000 [8]	Infected root canals (2)	Putative
*Pauljensenia hongkongensis*	2003 [82]	Pus/pelvic actinomycosis (1)	Putative
*Bowdeniella nasicola*	2003 [88]	Pus from nose (1)	Not determined
*A. oricola*	2003 [85]	Dental abscess (1)	Putative
*A. dentalis*	2005 [84]	Dental abscess (1)	Putative
*A. johnsonii*	2009 [83]	Subgingival plaque (2)	Putative
*A. massiliensis*	2009 [86]	Blood (1)	Putative
*A. timonensis*	2010 [87]	Osteoarticular sample (1)	Putative
*Gleimia hominis*	2010 [81]	Wound swab (1)	Putative

^a^the number of strains included in the description.

## Summary

As is the case especially in polymicrobial infection, both clinicians’ awareness and the microbiology team’s input are advantageous to detect the causative organism(s) responsible for the condition. Availability of advanced methods, such as MALDI-TOF MS and partial 16S rRNA sequencing, in clinical microbiology laboratories enables an accurate identification of *Actinomyces*-like isolates from patients’ samples, thus gradually elucidating their emerging role in clinically relevant infections. Further increasing of the number of strains in the database of MALDI-TOF MS, however, is necessary to improve the species-level identification rates of published but clinically less-known members of *Actinomyces* and newly reclassified related genera. *Actinomyces* and *Schaalia* species, in particular, can be found among polymicrobial consortia in infections at a variety of body sites. Also, the species *G. europaea* and *W. neuii* within the novel genera *Gleimia* and *Winkia*, respectively, possess an increased clinical significance in various types of human infections. The common presence of *Actinomyces* and related taxa in polymicrobial infections may be due to their contribution to pathogenic processes infecting humans. Increasing knowledge of these established and putative pathogens would bring about improved outcomes for patient care.

## Data Availability

Data sharing is not applicable to this article as no new data were created.
